# Supplementary figures and images for: Targeted detection and quantitation of histone modifications from 1,000 cells
Source: PLoS One. 2020 Oct 26;15(10):e0240829. doi: 10.1371/journal.pone.0240829 (PMC7588077; doi:10.1371/journal.pone.0240829)

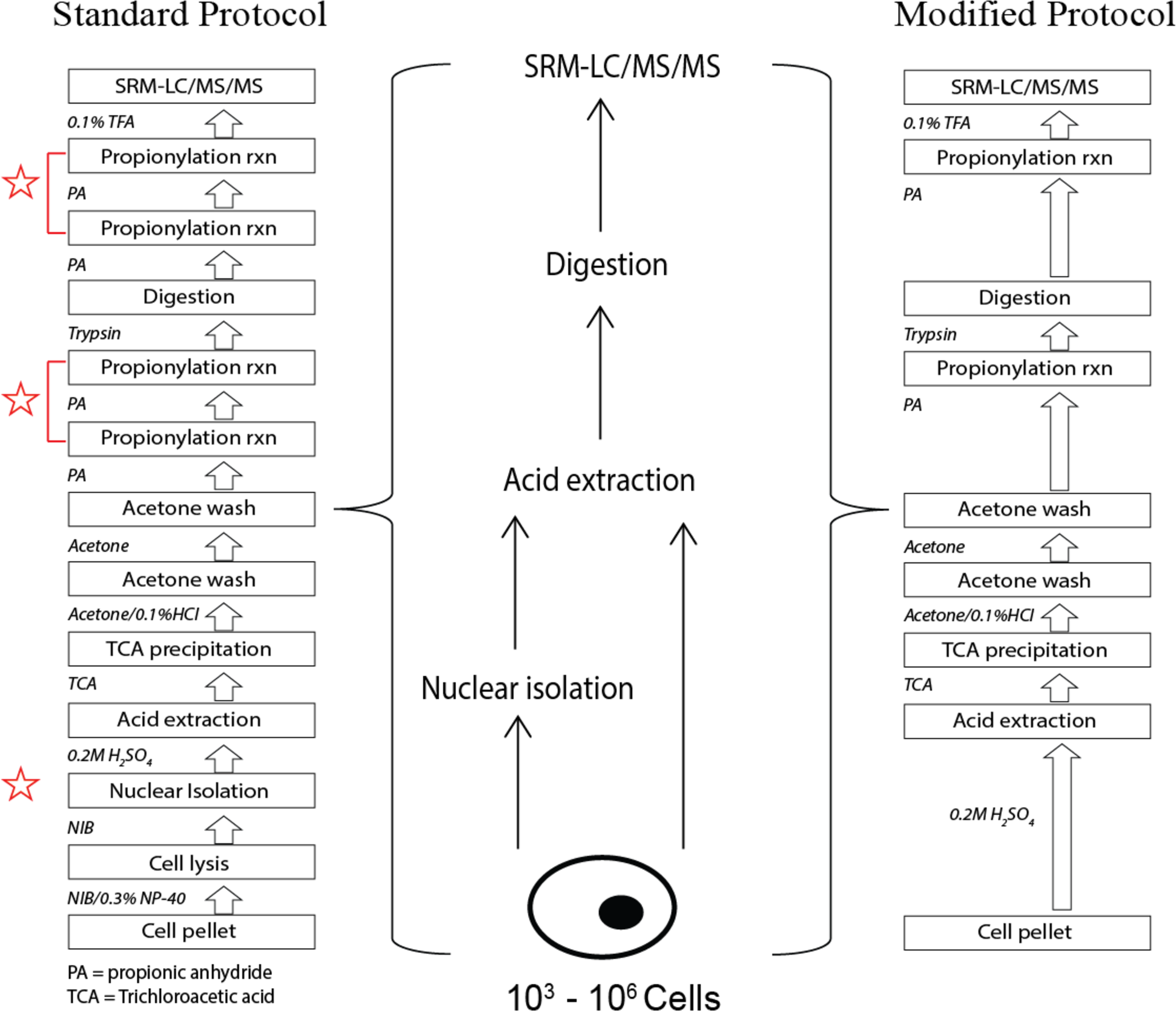

Supplement: S1 Fig — (TIF) [file pone.0240829.s001.tif]

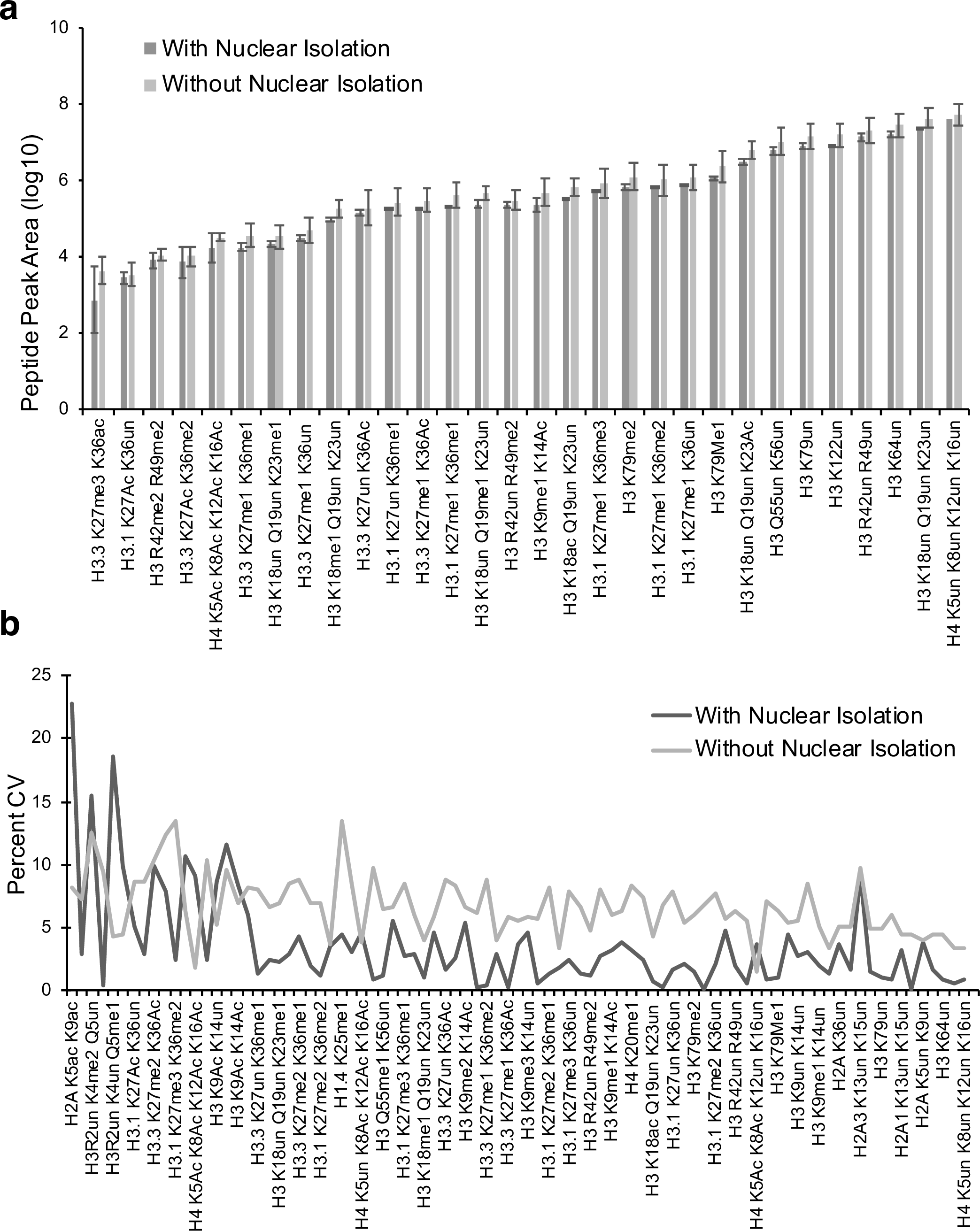

Supplement: S2 Fig — Comparison of (A) extracted peak area of histone peptides and (B) percentage CV values of histone peptides from 5×10^4^ cells with and without the nuclear isolation step. Error bars represent the standard deviation of two instrument replicates within a single experiment. (TIF) [file pone.0240829.s002.tif]

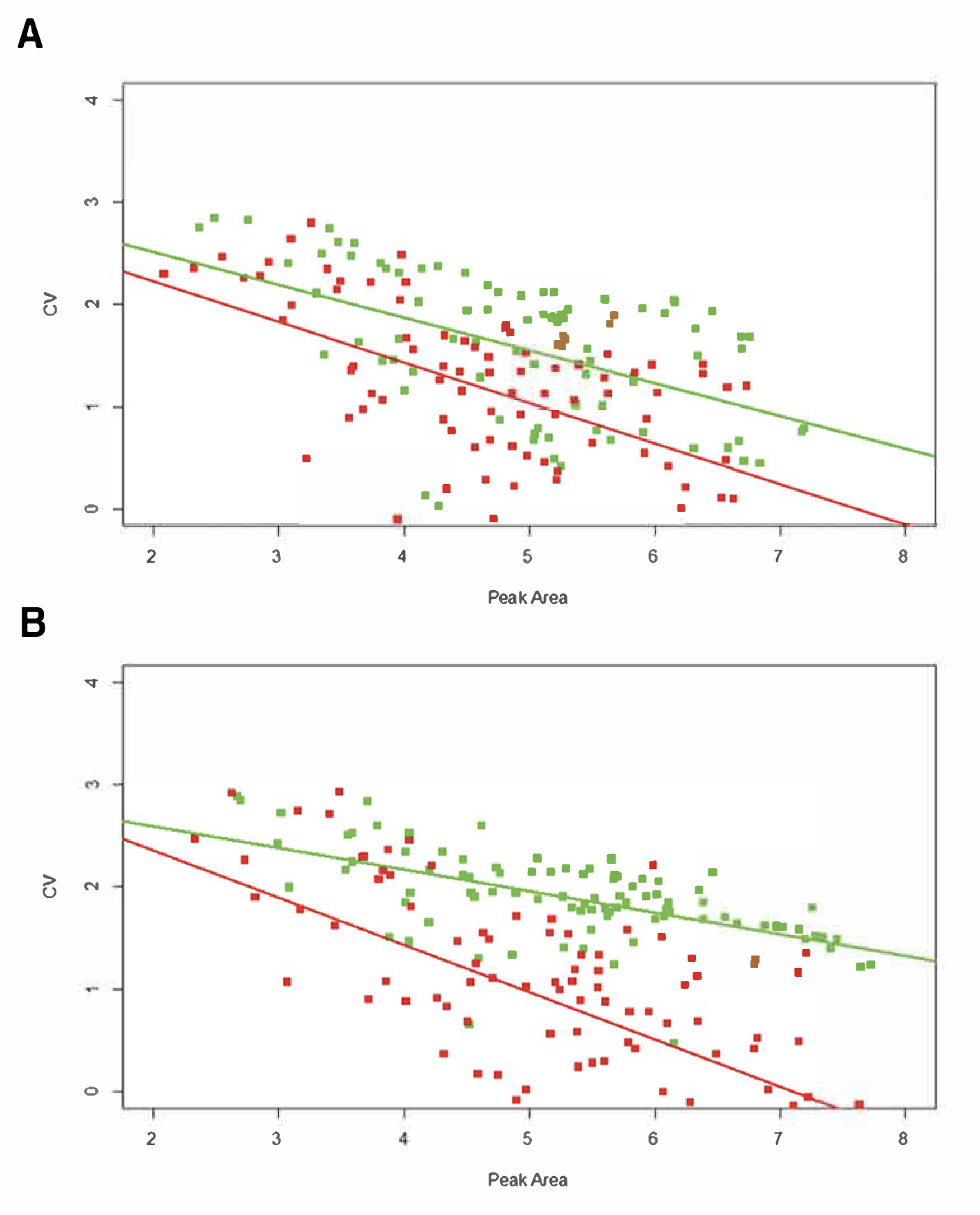

Supplement: S3 Fig — Log10 of peak area for each cell number was plotted with log10 of the coefficient of variation (CV). (A) 1×10^4^ cells show the same slope with (red) and without (green) nuclear isolation and a statistically significant difference in the lines (p = 6.055e-6), but not the slopes (p = 0.4254) by analysis of covariance (ANCOVA). (B) 5×10^4^ cells show different slopes with (red) and without (green) nuclear isolation and a statistically significant difference in the lines (p<2.2e-16) and slopes (p = 0.001876) by ANCOVA. (TIF) [file pone.0240829.s003.tif]

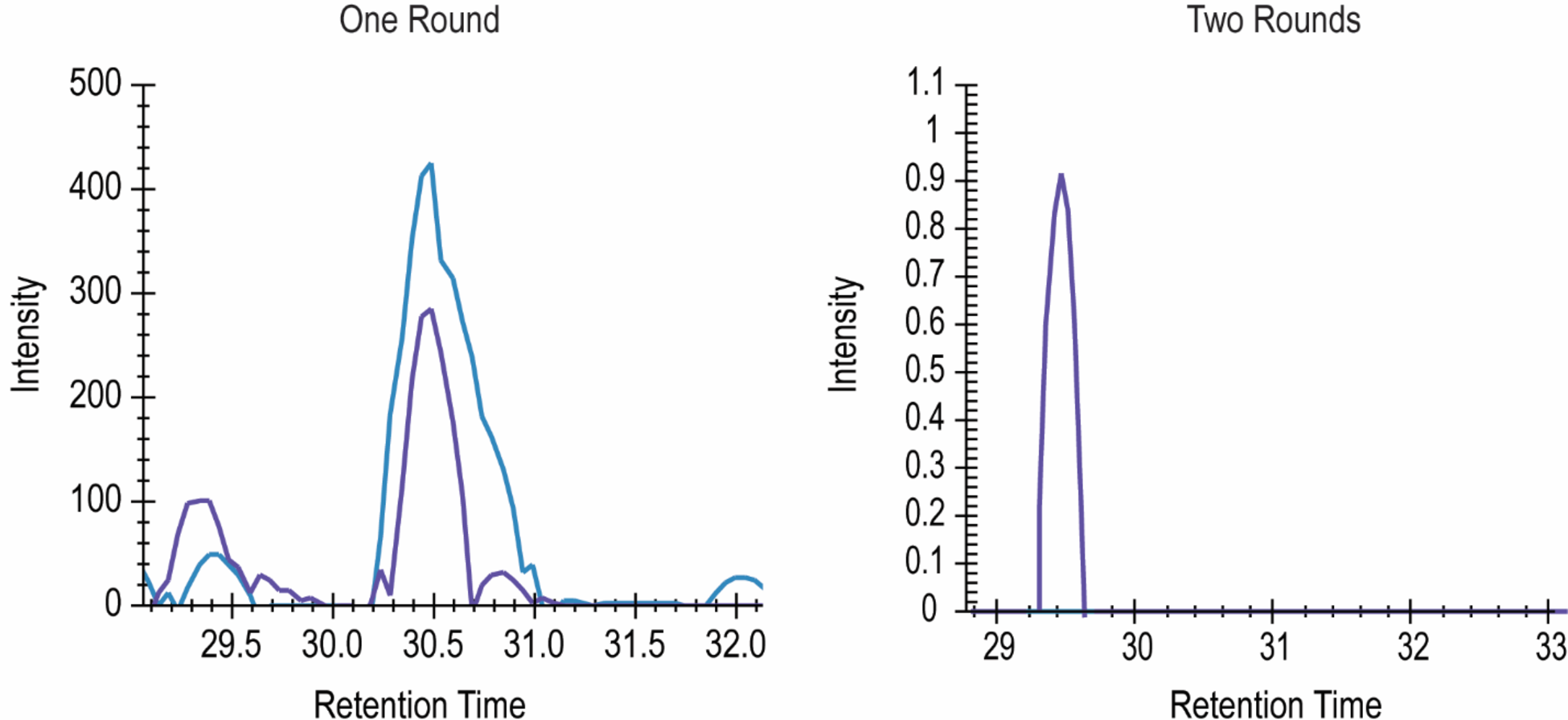

Supplement: S4 Fig — Skyline extracted peaks for H3.3 K27ac K36un shows the presence of the peptide in one round of propionylation and absent in two rounds. (TIF) [file pone.0240829.s004.tif]

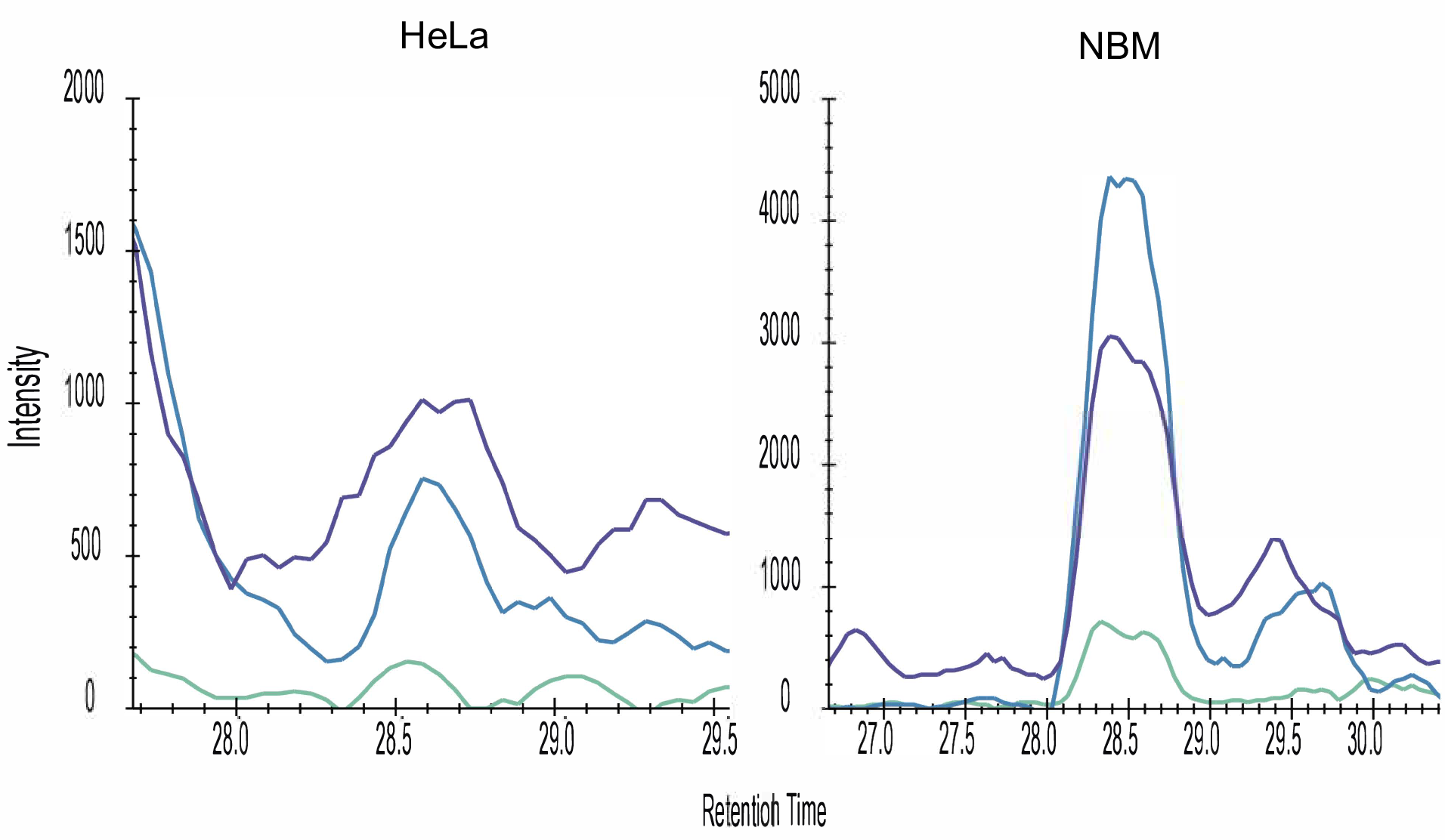

Supplement: S5 Fig — Comparison of H3 K9ac K14ac peptide in HeLa (left) and NBM (right) shows the presence of the peptide in NBM but not HeLa. (TIF) [file pone.0240829.s005.tif]

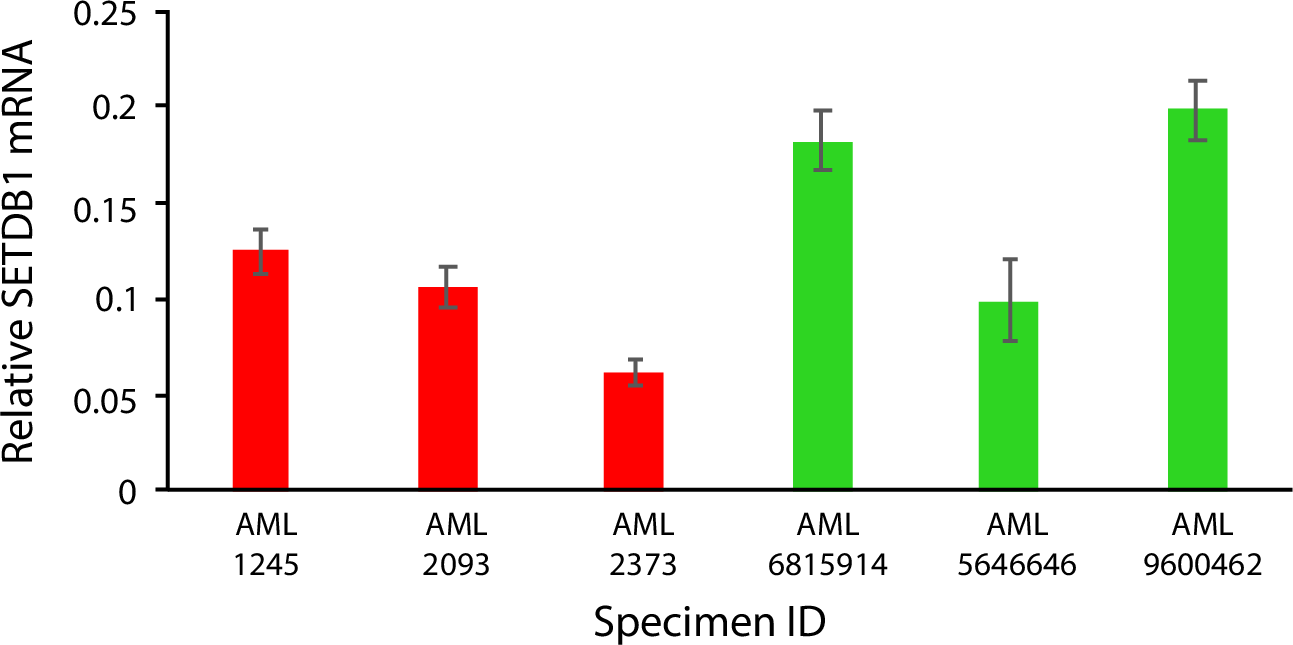

Supplement: S6 Fig — Relative SETDB1 mRNA levels as determined by qPCR in three samples with low H3K9me2/3 (red) and three samples with high H3K9me2/3 (blue). (TIF) [file pone.0240829.s006.tif]
