# Supplementary material for: Targeted detection and quantitation of histone modifications from 1,000 cells
Source: PLoS One. 2020 Oct 26;15(10):e0240829. doi: 10.1371/journal.pone.0240829 (PMC7588077; doi:10.1371/journal.pone.0240829)
Supplement: Attachment — Submitted filename: 20200622_PLOSone_Abshiru_Reviewer_Response_JMC_v2.docx [file pone.0240829.s014.docx]

We would like to thank the reviewers for their thoughts, comments, and suggestions on this manuscript. Responses are in bold throughout.

Review Comments to the Author

Please use the space provided to explain your answers to the questions above. You may also include additional comments for the author, including concerns about dual publication, research ethics, or publication ethics. (Please upload your review as an attachment if it exceeds 20,000 characters)

Reviewer #1: The paper entitled "Targeted detection and quantitation of histone modifications from 1,000 cells" from Kelleher’s group is an interesting and well-planned research on the improvement of sample preparation for histone PTM study. The manuscript is good as for contents, methodology and data analysis, the writing style and the clarity of the exposition are accurate. Conclusions are clear and Ref list is complete and correct. I have no specific concern but it is mandatory to explain/correct the manuscript in some points:

· Fragment type (CID or HCD) should be provided in the method part.
· Authors claimed that serial dilutions were used to make sure HeLa-S3 cells down to 1×103 cells. Why don’t use flow cytometry here?
· Heatmap in the Figure 5A is missing. b and y ions should be labeled in the Figure 5B.
· b and y ions in the Figure 4- are these from unique peptides? Any evidences to support the localization of Me/Ac on the K? Representative MS2 should be provided here.

**We appreciate the suggestions from the reviewer.**

1. **The fragmentation type has been added to the methods (pg 7, line 155).**
2. **As for the use of serial dilutions rather than flow cytometry, the cells were not grown in culture in house and were already stored as dry pellets prior to resuspension and dilution. We published on the use of flow sorted peripheral blood mononuclear cells at 100,000 cells per sample (J Am Soc Mass Spectrom. 2019 Dec;30(12):2526-2534), and this protocol could be readily adapted to accommodate 1000 sorted cells.**
3. **We apologize for the incomplete figure. The correct figure has now been included.**
4. **The b and y ions in the figure represent the ions that are monitored in the multiple reaction monitoring method. These ions were selected to unambiguously confirm the site of modification. The type of modification (Me/Ac) is determined by the mass and retention time of the precursor ion. As these data are collected in a multiple reaction monitoring manner, full MS2 fragmentation spectra are not available.**

Reviewer #2: The author in this paper primarily demonstrated the detection of histone peptides from limited number of cells using a targeted LC-MS/MS approach. This paper is interesting and certainly has potential applications towards proteomics at few or even single cell level. Combined with previous publications such as Luo et. Al., Anal. Chem. 2017, 89, 21, 11664–11671, it becomes feasible to pursue multi-omics research with under 1000 cells, which will potentially resolve unique properties of rare population of cells. In this regard, I think this paper is significant, and meets the journal standard for publication, although it lacks originality (i.e., modified protocols are reported).

Additionally, it would be helpful for the author to have a discussion on how this work relates to current multi-omics research, and further demonstrate its capability to study particular type of cells of interest.

**We appreciate the positive remarks from the reviewer. We have included additional discussion as to how this work fits into the field of multi-omics research (pg 15, line 331).**

Reviewer #3: In this manuscript Abshiru et al. present work that improves the sensitivity on a number of cells analyze basis for proteomic quantitation of histone PTMs. The key step is skipping nuclei isolation for cell numbers less than 1x10^4 and disposing of additional rounds of derivitization. This makes significant advances in sensitive histone proteomic analysis for limited samples in a variety of applications. The overall quality of the work is very good if spotty in places and I am confident in its publication in the near future. There are a few issues that unfortunately preclude its expedited publication without an additional round of review.

Major issues:

1) Figure 5 is missing and has not been peer reviewed. This is the crucial data conveying any actual biological insight. These results are also poorly described in text and no statistical analysis is presented for the interpretation and focus on specific results.

**We apologize for the omission of this figure in the original submission. It has been included in this revision.**

2) The manuscript fails to put this work in proper context from an analytical perspective. The improvement is compared relative to a 13 year old paper with no mention of improvements or comparison to recent work.

**We have included additional discussion of the analytical advancement of this paper in the discussion (pg 15, line 321).**

3) The discussion of the biological results is near-completely absent and is lacking context as well.

**We have added additional discussion on the biological results for AML samples (pg 16, line 344). We refrained from overstating biological interpretations and implications of these data given the limited specimens profiled. A deeper analysis of the histone PTMs and their association with AML biology would require a much larger cohort of specimens, given the disease heterogeneity, and is therefore outside the scope of this work.**

4) There are some major issue around the statistical interpretation of figure 1, given the apparent use of instrument technical replicates on single samples to infer significance of differences between the workflows.

**We regret the lack of clarity for Figure 1.** **These data were acquired in biological triplicate with a single technical injection. The text for the figure legend has been revised (pg 10, line 212) and the raw data have also been added to Table S1. Significant differences can be inferred by biological triplicate.**

Statistics:
The authors are generally clear and transparent about the use of statistics; however, there is some questionable apples-to-oranges interpretation of the statistics.

In text comments:

88
“However, this methodology is not applicable for the study of clinical samples with limited cells.” – This claim is overstated. A million cells or even a few million cells are a feasible number for many clinical applications. Yes, this is true for some, or even many, clinical applications as well as some basic science questions. Certainly, sensitivity gains are broadly useful.

**We agree that there are many circumstances where the number of cells is not a challenge for routine analysis. However, there are circumstances, such as investigation into leukemia stem cells, which would not be amenable to prior methodology.**

202
The data does not support these statements. Unless I missed something, the variances used for the statistics are technical and thus do not infer a statistically meaningful difference between the workflows. These results say that your instrument variance is smaller than the difference between these two samples. Repeating the workflow to measure the workflow variance would be necessary to make these statements. I think you could make more general claims of similarity. They certainly look similar and the data has not been obviously compromised by the new workflow.

**We apologize for the lack of clarity in the number of biological and technical replicates. All data were acquired with at least 3 biological replicates. Only samples acquired at 10^6^ cells were acquired with technical triplicate for each biological replicate. The averages shown represent the average of biological replicates. We have included additional wording as to the number of replicates in the Methods section (pg 8, line 162)**

219
“The standard histone propionylation protocol is time-consuming, typically requiring multiple days of sample preparation.” – Days, really? This only takes minutes of actual work and perhaps a few hours overall. Perhaps you are referring to from cells to the mass spec?

**Yes, we are referring to the complete procedure, from starting material to when the peptides are ready to be resuspended and placed on the instrument. We have changed the wording of this sentence to better reflect the complete sample preparation process.**

258
The use of the word “asses” is inappropriate.

**We apologize for the typo. The correction to “assess” has been made in the text.**

274
“Histone PTMs’ assessment in 1×106 cells was performed in six patients.” – Rewrite this sentence

**This sentence has been re-written as “We used the revised protocol to determine the relative abundance of histone PTMs in six AML patients at 1×10^6^ cells” (pg 13, line 275).**

275
“We identified consistent levels of H3 K9me2 and H3 K9me3 between two patient groups (Fig 5A and S5 Table).” – Figure 5 does not appear to be the correct figure to cite here. Also this sentence is followed by “We considered the possibility that the difference in H3 K9me levels could be…” The meaning of these two sentences is unclear. “consistent between two patient groups” and “the different in…” do not follow each other. Certainly there is some point that is not clear. This is a significant crux of the paper regarding any biological insight and it is completely unclear.

**We apologize for the incomplete figure. The correct figure has now been included. Of the two patient groups (N=3 for each), H3K9me2/3 was consistently high in one patient group and consistently low in the other. The phrasing of the text is poor. We have edited the text to reflect this difference, “Levels of H3K9me2 and H3K9me3 were high in a subset of three AML patients while low in the second subset of three AML patients (Fig 5A and S5 Table)” (pg 13, line 276).**

286
Again, there is a misplaced figure. I see no heat maps in any of the main figures. This makes it hard to review the results.

**We apologize for the incomplete figure. The correct figure has now been included.**

291
Table 1 is titled as “identified” but the caption calls them “quantifiable”. There is a difference.

**The title has been corrected to “quantified.” All noted peptides were considered quantifiable with a signal-to-noise ratio of 3.**

299
The paper cited is certainly important. It is an early effort that made important methodological innovations for histones. However, the use of the Gold standard moniker here seems misguided. Perhaps it is in the sense that it set a marker down of 1ug and is a good point of comparison in the distant past. Making a comparison to this work with respect to sensitivity is misleading. Dramatic improvements in sensitivity have been made in the intervening 13 years. Notably the introduction of the one-pot approach just a couple years later, which skips the HPLC purification step and analyzes the entire acid soluble fraction collectively, as is common today, dramatically improved the sensitivity. (J Proteome Res. 2009 Nov;8(11):5367-74) Instruments have also improved in the intervening years. It is true that good metrics of sensitive and efficient analysis are frequently notably absent there are a few more recent papers that are good points of comparison. There is already work down to 50,000 cells (Journal of Proteome Research, 20 Nov 2017, 17(1):234-242) and they did perform nuclei isolation. However, they do not state the amount loaded on column directly; however, there is recent top down work on histones the clearly states injecting 55 ng on column (J. Am. Soc. Mass Spectrom. 2019, 30, 12, 2548–2560). Clearly, the number to compare to in a modern context is much lower than 1µg. Also clear from looking for good points of comparison is that more work is needed that endeavors to bring these numbers further down with clearly defined metrics. Your work clearly does this but could use well researched context. It is your position as author to do this sort of research and educate the reader. As you fairly point out there is also a difference between on column sensitivity and the sample requirements.

**We do not intend for the “gold standard” to represent the best sensitivity, but rather the sample preparation protocol has been deemed the best for histone preparation and analysis by bottom-up mass spectrometry. The majority of histone MS publications, including the ones you list above (J Proteome Res. 2009 Nov;8(11):5367-74 and J Proteome Res. 20 Nov 2017, 17(1):234-242) employ the original histone double derivatization-digestion-double derivatization method from Garcia et al. 2007, though, as your state, histones are commonly analyzed together rather than individually isolated. Indeed, improvements in sensitivity have occurred over the past 13 years in regard to instrumentation. This does, however, make the sample requirement more challenging, as different instruments and different instrument methods will have different sensitivities.**

325
This is an incredibly sparse discussion of the AML results. There is plenty of context to give here. It does not need to be really big but this is almost nothing.

**We have added additional discussion regarding the AML results (pg 16, line 344).**

Figure 1
The use of instrument technical variance does not support there being any statistical significance to the difference between with and without nuclei isolation, which is the point of this figure to my understanding.

**We apologize for the inaccuracy of the figure legend. These data were acquired in biological triplicate with a single technical injection. The figure legend has been revised to reflect this information on pg 10, line 212. The raw peak area values used for the generation of these data have also been included in Table S1, tabs “10k” and “50k.”**

Figure 3
The figure would be clearer with additional labels on A and C.The figure caption is slightly confusing primarily due to the organization and labeling. although I think I figured it out. I might go with just an A panel (for A & B) and a B panel (for C & D) if you are not describing the panels separately.

**We have changed this figure to simply panel A (for A and B) and panel B (for C and D) based on the reviewer’s suggestion.**

Figure 4
The caption oddly starts out on the very general subject before describing what the figure is.

**We have modified the text of the figure legend on pg 12, line 270.**

Figure 5
Wrong figure included in file. This is a critical figure and I do not have access to critically review it.

**We apologize for the incomplete figure. The correct figure has now been included.**

Supplemental figure 1
It seems to me that this should be Figure 1 and not a supplemental figure. At the least it would be useful and informative to compare your results to previous work in the area of Leukemia and Histones PTM quantitation, particularly in vivo: (e.g. Blood (2014) 124 (21): 2202 & Blood (2019) 134(24): 2183-2194) or even in vitro J. Am. Soc. Mass Spectrom. 2004;15(1):77–86. Has this (*whatever this is* since the figure is missing and the results poorly described) been observed previously or is it novel. Does SETDB1 have an established role in leukemogenesis? A quick google search is a resounding yes, yet no mention here. I infer from Fig S6 that you probably observed higher H3K9me3. Contrary to the SETDB1 story I also see in the literature this biology is mostly connected with higher levels of acetylation, including K9ac which block SETDB1 activity and is antagonistic of K9me3. How to rationalize these.

**We have included additional discussion on the AML data in both the results (pg 13, line 276) and discussion (pg 16, line 344). The papers you list do not provide an adequate comparison with our data, as they were looking at changes in histone modification as a result of perturbation in the system being investigated (e.g. looking at changes in acetylation following disruption of acetyl-CoA as in Blood (2019) 134(24): 2183-2194). The majority of histone modification studies in AML focus on specific mutations or dysregulation of enzymes, such as those with EZH2 (Leukemia (2014) 28, 44–49**) **and DOT1L (Blood (2013) 121(13):2533-41). Given the lack of genetic information on the AML samples, it is difficult to generalize these results to results obtained with specific AML genetic backgrounds.**

There are a number of citations that worthy of inclusion (incomplete list):

From the Garcia Lab:
One-Pot Shotgun Quantitative Mass Spectrometry Characterization of Histones. J Proteome Res. 2009 Nov;8(11):5367-74
Dramatically improved sensitivity and established the practice of skipping further purification after dervitization, which is an integral part of your approach. Your work is an extension of gaining sensitivity by reducing steps.

From the Turner Lab:
Reading Signals on the Nucleosome With a New Nomenclature for Modified Histones. Nat Struct Mol Biol. 2005 Feb;12(2):110-2. doi: 10.1038/nsmb0205-110.
This established the Brno nomenclature for histone modifications used throughout this work. It would be helpful to reference this at line 164.

The Tiziana Bonaldi lab has published extensively on clinical applications of histone PTM proteomics on limited samples, including human tumors etc.

**Thank you for the additional reference suggestions. We have included additional references to properly place this work within the field. As an aside, J Proteome Res. 2009 Nov;8(11):5367-74 from the Garcia lab did not dramatically improve sensitivity. Sensitivity was deemed comparable to the original work, which purified histones off-line by HPLC and analyzed each independently. The goal of the 2009 work was to increase throughput but analyzing all histones at once, as opposed to each histone faction individually. Throughput was further increased by labelling two different samples with deuterated or non-deuterated propionic anhydride, thereby allowing samples to be mixed and analyzed in a single run.**

**Regarding the SETDB1 biology, given the small number of specimens assessed, it is difficult to delineate a clear disease mechanism in AML from the data presented. We have included a reference (J Cell Biol (2017) 216(11): 3535–3549) which links H3 K9 methylation, SETDB1 expression and associated leukemia evasion of immune surveillance as one example where aberrant findings of these molecular parameters could suggest a biological mechanism of disease.**
