# Supplementary material for: Targeted detection and quantitation of histone modifications from 1,000 cells
Source: PLoS One. 2020 Oct 26;15(10):e0240829. doi: 10.1371/journal.pone.0240829 (PMC7588077; doi:10.1371/journal.pone.0240829)
Supplement: Attachment — Submitted filename: 20200826_PLOSone_Response_2_v2_PMT.docx [file pone.0240829.s015.docx]

Reviewer #1: Authors claimed that "these ions were selected to unambiguously confirm the site of modification". If MS2 spectra are not available, it would be good to provide solid evidence to support these ion transitions.

**Peptide transitions that were used in this manuscript were previously developed and published. Zheng et al. (Zheng Y, Sweet SM, Popovic R, Martinez-Garcia E, Tipton JD, Thomas PM, et al. Total kinetic analysis reveals how combinatorial methylation patterns are established on lysines 27 and 36 of histone H3. Proceedings of the National Academy of Sciences of the United States of America. 2012;109(34):13549-54) initially developed the methods for methylated peptides. One year later, he further exanded this to acetylated peptides (Zheng Y, Thomas PM, Kelleher NL. Measurement of acetylation turnover at distinct lysines in human histones identifies long-lived acetylation sites. Nature communications. 2013;4:2203).**

**We have included these references within the methods on pg 7, line 157, as well as a full list of all transitions as a supplemental table (S7 Table).**

Reviewer #2: Discussion of multi-omics approaches are added in this version, and I believe it is suitable for publication.

Reviewer #3: In this revised manuscript Abshiru et al. present work that improves the sensitivity on a number of cells analyze basis for proteomic quantitation of histone PTMs. The key step is skipping nuclei isolation for cell numbers less than 1x10^4 and disposing of additional rounds of derivitization. This makes significant advances in sensitive histone proteomic analysis for limited samples in a variety of applications. The overall quality of the work is very good and the sensitivity improvements are substantial. The improvements presented here are significant and important; however, the claims remain somewhat overstated and poorly contextualized. The responsiveness of the authors to some reviewer comments was limited and half-hearted, in other places the response is thorough and appropriate. Many major errors and mistakes in language have been fixed. A major flaw remains in the apparent misreading or possible misrepresentation of the prior literature and the overstatement of the improvements made. I find the response of the authors to the reviewer comments on this subject perplexing bordering on disconcerting. I see no need to misrepresent the value of the current work by creating a straw man when the improvements offered in the current work are real and significant when compared to reality.

**We thank the reviewer for their comments on the strong quality of the work submitted. We certainly didn’t intend to offend, misrepresent or overstate the significance of the work submitted and had believed that we had fully addressed these issues in the prior revision. To address these lingering concerns, were have re-written the discussion section to include additional comparisons to the current literature and hope this will now meet the needs of the reviewers and editors of PLoS One.**

Major revision

Crux of the major issue:

You completely misread the Guo paper: The claim of the Guo paper is that the prevailing methods (dating back to ~2009) ARE capable of obtaining results from 50,000 cells NOT that they have an improved method that is capable of such. This evidence is clear and unambiguous.

**As discussed above, we do not dispute this fact and have re-written the discussion to include a more in-depth comparison with the current histone sample preparation literature (pg. 15, line 336).**

Comments on prior literature generally:

Certainly, one of the issues is that the prior literature has not been as careful or consistent in presenting quantitative metrics of sensitivity and sample requirements. Sometimes specific sensitivity claims are not even stated while other use differing metrics. This makes proper presentation of the context an effort; however, the authors should make the effort and interpret the literature for the audience. The difference between the “number of cells” and “mass of protein on column” sensitivities is important to this particular effort. The frequent but not universal absence of solid sensitivity claims in prior work should not justify claiming that there are no improvements but rather noting this fact prominently as incomplete information in the literature and that it may be hard to judge. A little reading allows fairly clear inferences about prior work. For example, the Guo paper that is now cited for the clear and comparable “50,000 cells” claim includes this sentence: “Histones were extracted as described previously with minor modification” and cites a Jove paper which is essentially a video version of the 2009 J Proteome Res. One Pot paper. Thus, they are the same procedure with inconsequential modification. It is quite clear from reading the Guo paper carefully that they are not presenting an improved method that is now capable of this. The very title begins with “Assessment of …”. Thus, it is claiming that this is the capacity of the established methods but no one has ever really assessed this capacity properly. “…our study addresses a simple, albeit critical, question about the amount of material required for MS analysis of histone PTMs.” Lots of papers in this area start with large numbers of cells not because it is a requirement but because it is easy, convenient and the work is not sample limited (cell culture or tumors). It is also obvious that skipping the HPLC step as was done in the one pot paper was essential to this sensitivity capacity demonstrated by Guo. This is in fact the primary thesis of the improvements in this work as you state: “reduction in sample processing allows for quantitation of very low input samples.”. Skip nuclei isolation. Skip the second round of propionylation. Even more histones are extracted for low cell counts. There is no doubt that the contribution you are making here is important but do not overstate it. You have made a 5-fold to maybe approaching 50-fold improvement over the established methods that have been in use for over ten years, if only recently assessed for LLOQ by input cell number and not widely used in cell number limited work. Nonetheless, the improvements made here are pretty substantial and enabling of important future work.

**In the discussion, we have attempted to place our work within the context of what was previously done. With regard to the comparison with the Guo paper, we have stated that the results obtained at 50,000 cells when the nuclear isolation is omitted shows no benefit in sensitivity. For 50,000 cells and above, there is no benefit to our revised method and we suggest that the traditional approach may be preferred at these higher cell starting amounts (pg. 16, line 364).**

AML and biological context:

I agree entirely that care should be applied to not overstating the meaning of the data presented here in understanding the fundamental mechanisms of AML. There is simply not enough statistical power for such claims. However, there are trends that are present and worthy of discussion and comparison to other studies. The concordance with prior work is validating some of which have solid in vivo models, Kaplan-Meier curves and other follow up experiments from the proteomics data. It is also important to discuss the limitations of the data and warn readers not to over interpret it. The concordance of your (limited) results with (limited) prior observation are also validating of the capacity of your improved approach to accurately measure.

**Given the small “N”, we have been extremely careful not to overstate the findings in the context of AML. In this revision, we have expanded on the role of histone modifying enzymes in blood cancer beginning on pg 16, line 375. We have further shown evidence of the role of H3 K9me3 and SETDB1 levels in AML beginning on pg 17, line 402.**

References cited:

Separately from misrepresentation of the literature, the authors also seem to have a strong self citation bias and a tendency toward selective exclusion of inconvenient but appropriate literature for comparison.

**We respectfully disagree that we displayed “strong self-citation bias”. Our research group has been active in histone analysis for over 15 years, publishing over 50 papers in this field, much of it building on past methods developed by our group and others applying it within biological contexts. In the previous revision, just 6 of the 40 total references (15%) were to our prior work. With the additional references added in this revision, this ratio drops to 6/45 total or ~13%.**
