# Supplementary material for: Targeted detection and quantitation of histone modifications from 1,000 cells
Source: PLoS One. 2020 Oct 26;15(10):e0240829. doi: 10.1371/journal.pone.0240829 (PMC7588077; doi:10.1371/journal.pone.0240829)
Supplement: Attachment — Submitted filename: 20200826_PLOSone_Response_3_v1_JMC.docx [file pone.0240829.s016.docx]

The authors have addressed all the reviewer concerns. The new discussion is well presented.
There is one minor point: On page 25, line 530: There should be a table S7 listed along with its title.

**We appreciate the positive response to the revised manuscript. We have included the title for table S7 on pg 25, line 526.**
